# Imaging findings of urosymphyseal fistulas

**DOI:** 10.1259/bjrcr.20210217

**Published:** 2022-11-01

**Authors:** Kunj Patel, Hayan Butt, Shlok Patel, Jacques Roux, Ashish Bhagat

**Affiliations:** 1 West Hertfordshire NHS Trust, Hertfordshire, United Kingdom

## Abstract

Prostate cancer accounts for 13% of all new cancer diagnoses in the UK. Urosymphyseal fistulas are a rare complication that can occur post-radiotherapy and surgery for prostate cancer. Patients often present with non-specific symptoms such as suprapubic tenderness, poor mobility, recurrent urinary infections, and difficulty passing urine. These can be difficult to diagnose clinically and extremely problematic and debilitating for patients. The management of these patients is often complex and requires input from urology, orthopaedics, and microbiology. At present, there are no clear guidelines for diagnosing these conditions. Recommended investigations include blood tests, urine culture, and imaging. The preferred imaging modality is pelvic MRI. This article explores three rare cases of such complications and the classic imaging findings on CT and MRI to aid the diagnosis of urosymphyseal fistula.

## Introduction

Prostate cancer is one of the most frequently occurring malignancies in males, accounting for 13% of all new cancer diagnoses in the UK.^1^ Curative treatment options for prostate cancer include radiotherapy and surgery. However, complications can occur following treatment with radiotherapy alone or in combination with surgery.^2^ These include the development of a fistula between the pubic bone and the urogenital tract, which can cause pubic bone osteomyelitis. Such complications, although rare, can be challenging to diagnose and extremely problematic and debilitating for patients. They can further increase the risk of morbidity and local complications such as pelvic pain and chronic urinary tract infections.^3^ This article explores three rare cases of such complications and the radiological features of urosymphyseal fistulae.

A diagnosis of urosymphyseal fistula or pubic bone osteomyelitis should be suspected in patients with a history of prostate cancer, managed with radiotherapy, surgery, or a combination of both. Presenting symptoms may include suprapubic tenderness, poor mobility, recurrent urinary infections, and difficulty passing urine.^4^ At present, there are no clear guidelines for diagnosing these conditions. Recommended investigations include blood tests, urine culture, and imaging. The preferred imaging modality is pelvic MRI. For patients unable to tolerate MRI scans, CT cystograms can also be used to identify fistulae, and cystoscopies are often necessary to evaluate bladder outlet dysfunction.^5^

The current body of literature is small, with less than 50 cases reported in the literature. Early diagnosis is crucial, and studies have shown that MRI is an excellent imaging technique to assess soft tissue-based pathology and has been used to detect urinary fistulae with good results.^6^ We present three rare cases of patients whose prostate cancer treatment was complicated by the development of urinary fistulation into the pubic symphysis post-radiotherapy. We discuss the classic imaging findings on CT and MRI in patients with prostato-symphyseal fistula (PSF) and associated complications.

## Clinical presentation and imaging findings

### Case 1

An 81-year-old male was admitted with severe lower abdominal pain with a 3 cm tender irreducible lump in the suprapubic region. He had a background of prostate cancer (T3b, Gleason 4 + 3 = 7) which was treated with radical whole pelvis radiotherapy and 3 years of androgen deprivation therapy. He subsequently developed bladder neck stenosis and bulbar urethral stricture 11 years post-treatment, for which he independently self-catheterised. Cystoscopy and cystolitholapaxy were also performed for bladder neck stenosis and bladder stones. Initially, he was referred to the surgical team and he had a CT abdomen and pelvis for a possible strangulated inguinal hernia.

**Figure 1.**
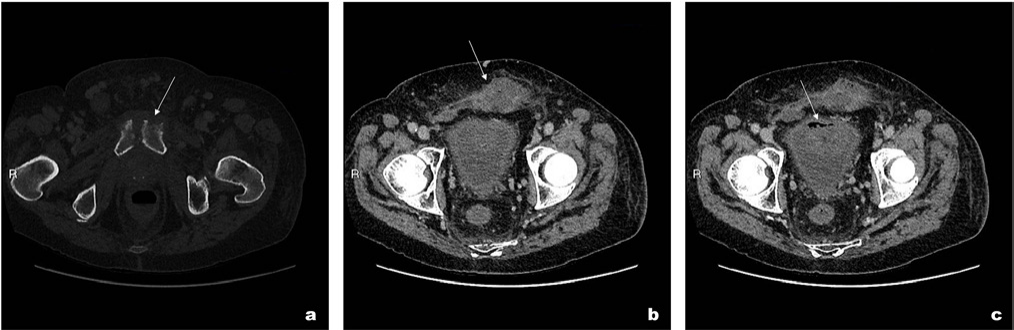
(a) CT axial (bone window) slice demonstrates irregular cortical bony destruction at the pubic symphysis with surrounding free fluid in keeping with osteomyelitis. (b) Axial slice (soft tissue window) in the portal venous phase demonstrate fluid collection within the anterior abdominal wall. (c) Thick-walled bladder with intraluminal air seen in the same window as Figure 1.

CT showed an irregular fluid collection with rim enhancement extending into the subcutaneous fat anterior to the bladder. Furthermore, cortical bone destruction was noted in keeping with pubic bone osteomyelitis (Figure 1a,b). Additionally, CT demonstrated a thick-walled urinary bladder with intraluminal air and mucosal enhancement (Figure 1c). The prostate was small and calcified. A possible diagnosis of cystitis was suggested from the CT findings.

**Figure 2.**
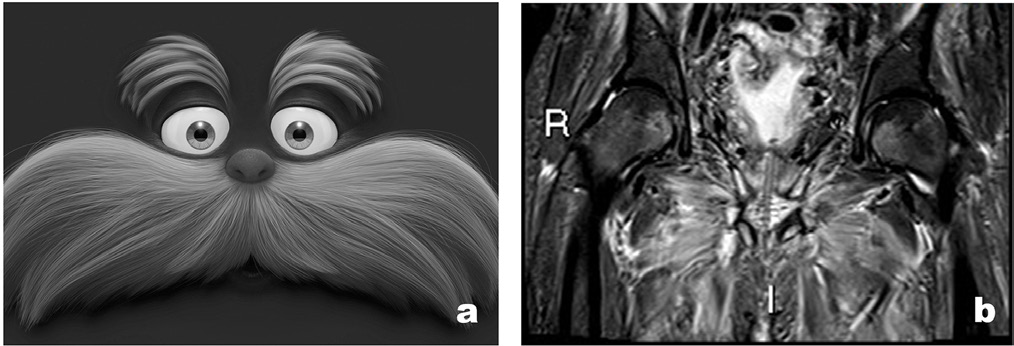
(a) Lorax character. (b) *T*_2_ weighted coronal MRI sequence showing symmetrical oedema involving the pectineus, obturators, and adductor muscles which resembles the appearance of Lorax.

The patient subsequently went to have an MRI scan for further evaluation. MRI showed low signal on T1 and high on T2 and STIR sequences in the pubic bone on either side of the pubic symphysis extending in the para-symphyseal musculature, including pectineus, obturator, and the adductor group. Moreover, MRI confirmed multiple soft tissue collections and abscesses (Figure 2). Post-intravenous contrast showed enhancement which was in keeping with osteomyelitis.

**Figure 3.**
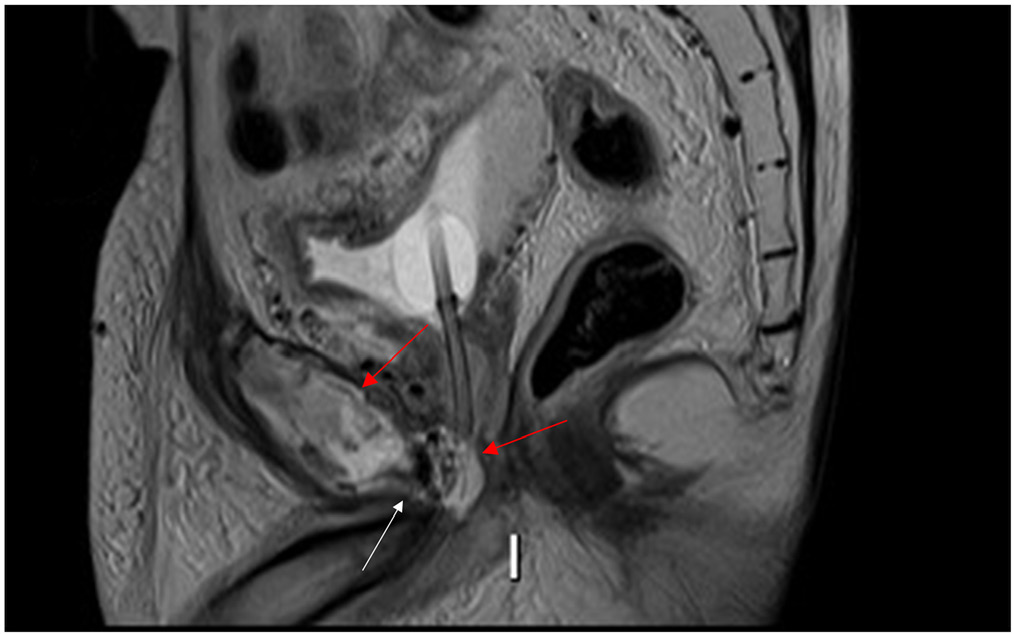
*T*_2_ weighted sagittal sequences demonstrate fistulous tract extending into the base of the penis (white arrow) with areas of enhancement within the region of the perineal body and pubic symphysis (red arrows).

These appearances were highly suggestive of a fistula from the prostate into the pubic symphysis giving rise to osteomyelitis and osteonecrosis. A closer review of imaging showed the fistulous tract extended into the base of the penis with areas of enhancement within the region of the perineal body seen in *T*_2_ weight sagittal sequence (Figure 3).

### Case 2

A 74-year-old patient presented with groin pain and a wound on his left upper thigh for 2 days with weeping yellow exudate. He had a past medical history of prostate cancer (Gleason 4 + 3 = 7, PSA 141, with no metastases) which was treated with radical external beam radiotherapy. The patient subsequently had LHRH androgen deprivation therapy. He also has a background of Type 2 diabetes, hypertension, and urinary incontinence.

**Figure 4.**
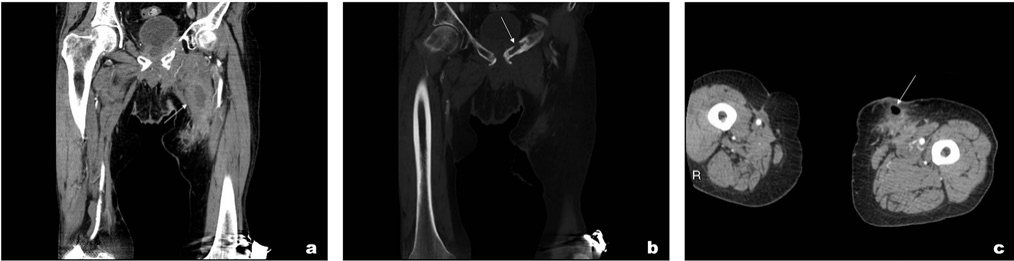
(a) Post-contrast CT coronal slice showing a thick-walled collection involving the left adductor longus & brevis with small fluid pockets surrounding the pubic symphysis. (b) Bony window shows destruction of the pubic symphysis. (c) Post-contract CT axial slice showing gas pockets within the subcutaneous tissue with surrounding fluid and fat stranding.

On presentation, he had elevated inflammatory markers (WCC 11.7, CRP 35) and subsequently had a CT abdomen and pelvis which showed a thick-walled collection (40 × 34 mm) involving the left adductor brevis muscle (Figure 4a). Furthermore, there were destructive changes involving the pubic tubercle and ramus bilaterally with surrounding free fluid and air pockets (Figure 4b,c).

**Figure 5.**
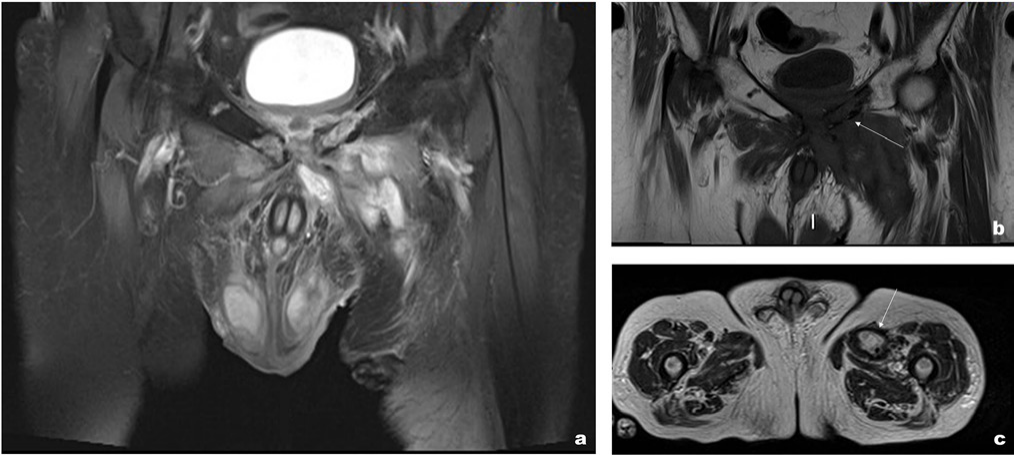
(a) Coronal *T*_2_ weighed sequence shows a high signal within the pubic symphysis in keeping with pubic bone osteomyelitis and multiple soft tissue collections involving the adductor muscles. (b) T1 weighed non-contrast coronal sequence showing low signal predominantly involving the left superior pubic ramus. (c) Axial *T*_2_ weighted sequence shows collection involving the left pectineus and adductor muscle groups.

The patient underwent an MRI without contrast of the left hip for further characterisation. The MRI showed pockets of collections along the left adductor muscle compartments extending into the deep pelvis with osteomyelitis involving the left pubic bone (Figure 5a). The adductor compartment and pubic bone osteomyelitis had low signal on T1 and high signal on STIR and coronal sequences (Figure 5b,c).

**Figure 6.**
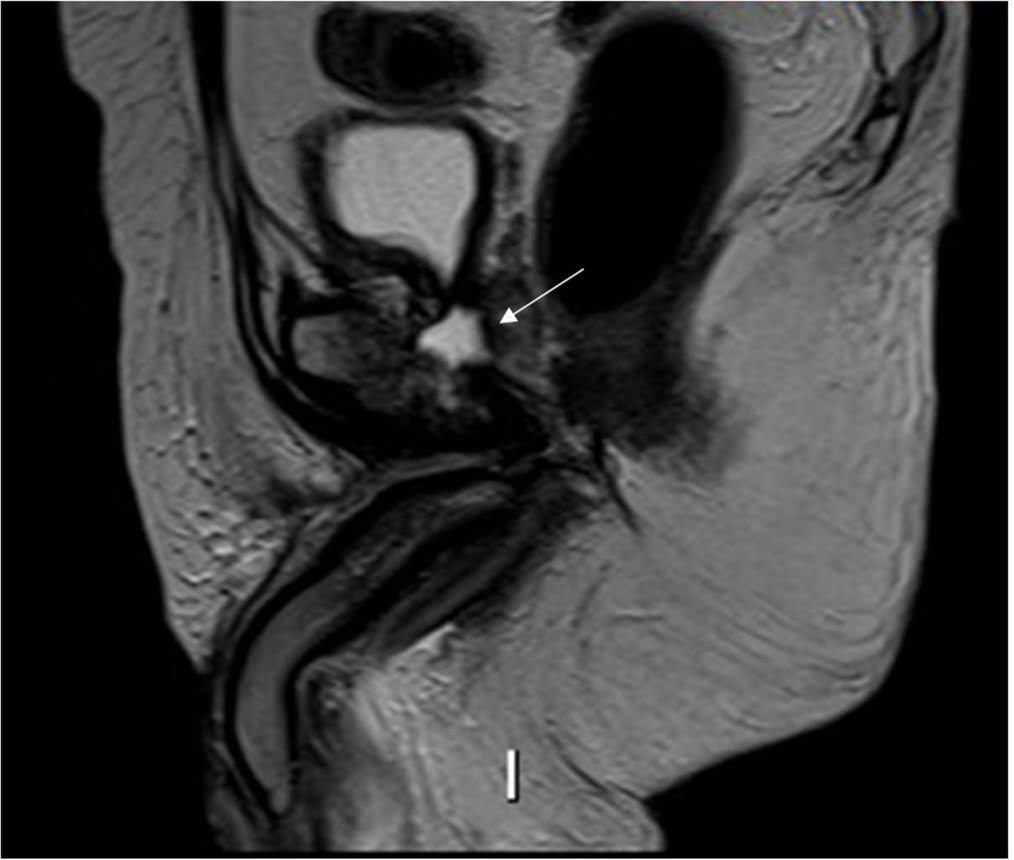
Sagittal *T_2_* weighted sequence shows a high signal within the pubic bone arising from below the neck of the bladder in keeping with urosympheseal fistula.

This was later rereviewed as per the clinical team’s request and a diagnosis of urosympheseal fistula was determined to be the underlying cause of collections (Figure 6). A urology opinion was recommended. The patient was given i.v. antibiotics as an inpatient and discharged with the view of having a long-term catheter and outpatient urology follow-up.

### Case 3

A 71-year-old male with a background of prostate cancer presented with a right groin lesion. The patient had a background of prostatectomy for prostate cancer with subsequent radiotherapy for recurrence.

**Figure 7.**
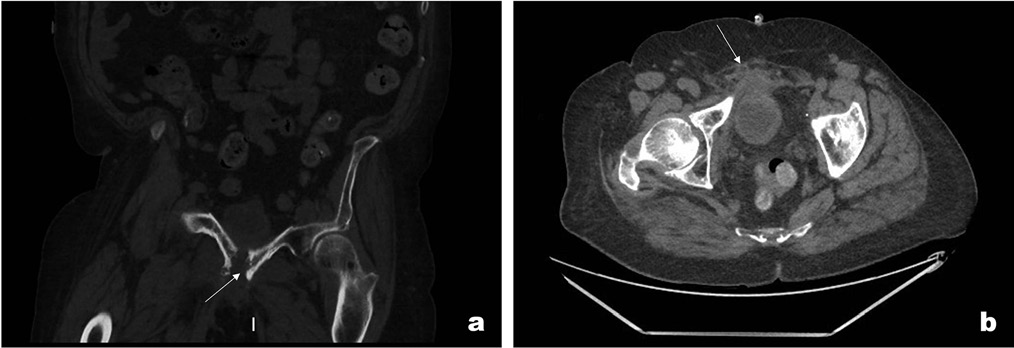
(a) Post-contrast CT coronal (bone window) slice showing cortical destruction involving the pubic bone bilaterally in keeping with osteomyelitis. (b) CT axial (soft tissue window) slices showing fluid surrounding the bladder and pubic symphysis with surrounding fat stranding.

**Figure 8.**
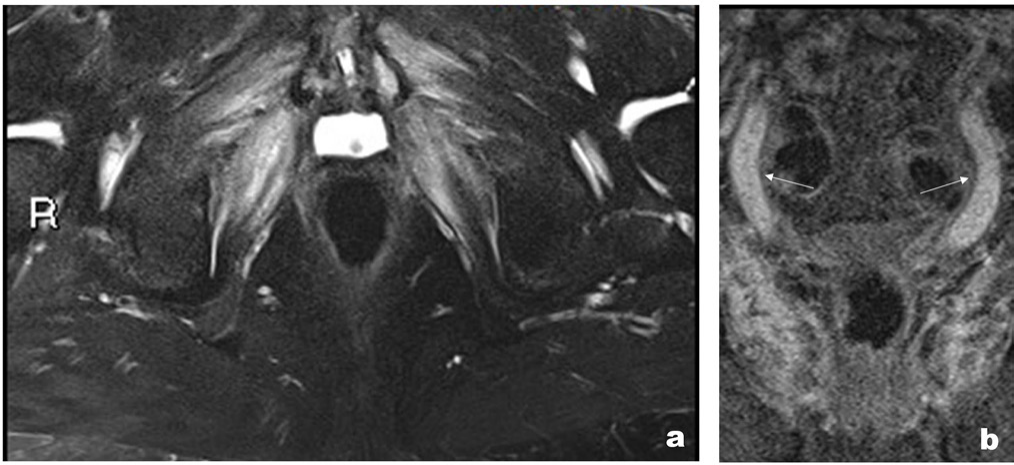
(a) MRI STIR axial sequence showing multiple fluid intensities involving the adductor muscles and the pubic symphysis in keeping with secondary osteomyelitis and myositis. (b) *T*_1_ weighted fat saturated coronal sequence showing bilateral hydroureters. STIR, short tau inversion recovery.

He initially had a CT abdomen and pelvis with contrast which showed multiple ill-defined areas of fluid collections surrounding pubic symphysis. His CT also showed evidence of osteomyelitis (Figure 7). Due to diagnostic uncertainty and his background of prostate Ca, MRI was performed. MRI showed a large fluid intensity tract extending from the perineum to the pubic symphysis, secondary osteomyelitis, and myositis (Figure 8a).

Moreover, there was a large tract extending from the skin of the right groin medially towards the perineum, bilateral hydroureter, and suprapubic catheter (Figure 8b). This confirmed the diagnosis of urosympheseal fistula. Since diagnosis, he has presented with recurrent collections and pubic bone osteomyelitis.

## Discussion

Pubic bone osteomyelitis with an associated urosymphyseal urinary fistula is rarely described in the literature with less than 50 cases reported. It is a rare complication of prostate surgery and radiotherapy. Recognition of clinical symptoms and imaging findings are crucial to establishing a diagnosis. Patients often present with symptoms that have wide differential diagnoses and often do not involve urology early on. In our experience, all patients were subsequently referred to urology after a diagnosis was made on imaging.

The pathophysiology of urosymphyseal fistula is unclear; however, it is becoming better understood through the analysis of patient cases. The development of urosymphyseal fistula following treatment for prostate cancer is believed to be associated with endoscopic treatments post-prostatectomy, transurethral resection of prostate, and radiotherapy.^2^ Surgery and radiotherapy increase the risk of stricture formation; hence endoscopic treatments such as cystoscopy are used to treat urethral stenosis and bladder outlet obstruction. However, this can inadvertently lead to urosymphyseal fistulation and subsequently pubic bone osteomyelitis as colonised urine is in contact with the pubis, enabling the transfer of bacteria.^2,7^ Pelvic radiotherapy can also increase the risk of osteomyelitis by altering wound healing.^3^ Other causes of pubic osteomyelitis include chronic indwelling catheters, photovaporisation of the prostate, and bone-anchored slings for stress urinary incontinence.^2^

Matsushita et al analysed 12 patients who developed urosymphyseal fistulae following prostate cancer treatment. Eight of the patients had primary radiotherapy (five of these eight which subsequently had radical prostatectomy too), while the other four patients in the study had radical prostatectomy followed by radiotherapy. All these patients developed bladder outlet obstruction which required endoscopic treatment. The median time between the endoscopic treatment and fistulae development was 36 months^7^. Furthermore, Bugeja et al analysed 16 prostate cancer patients who developed urosymphyseal fistulae, half of which had primary radiotherapy followed by radical prostatectomy, the other half had surgery first followed by radiotherapy. 13 of these patients had endoscopic or open procedures for bladder outlet obstruction, with subsequent urinary leak believed to be implicated in fistula formation.^8^ All patients in our case series had radical radiotherapy (one had a radical prostatectomy followed by radiotherapy) which would fit with the current literature.

The diagnostic imaging of osteomyelitis can require the combination of diverse imaging techniques for an accurate diagnosis which predominantly includes plain radiography, CT and MRI. Nuclear medicine techniques, although highly sensitive, are often non-specific. CT provides excellent multiplanar reconstructions allowing delineation of even the most subtle osseous changes. In chronic osteomyelitis, CT demonstrates abnormal thickening of the affected cortical bone, with sclerotic changes and chronic draining sinus. Although CT may show these changes earlier than plain radiograph, CT is less desirable than MRI because of decreased soft tissue contrast as well as exposure to ionising radiation.^5^

MRI is the most sensitive and specific imaging method as shown in literature and offers the advantage of being relatively less invasive than retrograde studies.^6^ It allows visualisation of fluid collections and the extent of osseous involvement and inflammatory changes. It often presents with a high signal on *T*_2_ weighted images and low signal intensity on *T*_1_ weighted images secondary to alteration of marrow signal intensity, due to underlying oedema. Some authors have identified the fistulous tract on heavily *T*_2_ weighted MR sequences.^9^ In our study, high T2 and STIR signal with low T1 signal was noted in all three patients on MRI. The literature suggests heavily weighted T2 sequences are most useful in these patients, however, this was not done in our centre and T2/STIR sequences were sufficient for diagnosis.^3,10^

If there are any signals of osteonecrosis or abscess formation, any gadolinium-based contrast would show further enhancement as seen in cases 1 and 2. Other MRI findings include cortical erosion with associated diastasis of the pubic symphysis cortical destruction, as seen in all our cases.^10^ In our experience, we often see symmetrical muscle oedema involving the adductor muscles, obturator internus and externus on either side of the pubic symphysis. All our patients presented with this appearance resembling Lorax and we would like to take this opportunity to name this the Lorax sign, inspired by the animated cartoon character of the movie Lorax. The symmetrical muscle oedema represents the moustache of Lorax and the obturator oedema corresponding to the eyebrows on coronal MRI sequences. Whilst this is not specific to PSF, we believe it is highly sensitive in patients with a background of prostate surgery and radiotherapy. Similar appearances can be seen secondary to osteomyelitis of the pubic bone and radiotherapy within the pelvis. Myositis from any cause can look similar, however, it would be unusual to only involve the adductor compartment.

The management of these patients is often complex and requires input from urology, orthopaedics and microbiology.^8^ Antibiotics are used to prevent urosepsis, however, conservative management alone is rarely successful, and surgery is often necessary.^8^ This typically involves resection of the infected bone, resection of fistulae and urinary diversion. The surgery performed will vary on a case-by-case basis and may involve a complete cystectomy with ileal conduit formation.

Surgical approaches have been shown to provide a statistically significant reduction in pelvic pain intensity in a study of 16 patients, compared to conservative measures.^11^ Of course, surgery is not without risk; one study investigating complications post-cystectomy found that substantial risks include intraoperative bleeding (mean blood loss 476 +_ 379 ml), post-operative ileus and infection.^12^ This highlights that this patient group requires strong rehabilitation in the post-operative phase. Although, Gupta et al 2014 found in a study of 10 patients with pubic osteomyelitis, that surgical management led to the complete resolution of symptoms; suggesting this is the optimal treatment option for this condition.^2^

## Conclusion

Due to the rarity of urosymphyseal fistulas, the diagnosis is often overlooked. Patients often present with non-specific symptoms and are referred to urology after imaging findings confirm the diagnosis. A diagnosis of urosymphyseal fistula or pubic bone osteomyelitis should be suspected in all patients with a history of prostate cancer, managed with radiotherapy or surgery. Reaching a diagnosis often requires the combination of diverse imaging techniques. CT imaging is useful to detect early osseous involvement in the presence of osteomyelitis. In all our patients, the definitive diagnosis was made with MRI imaging which has been described as the most sensitive and specific imaging modality in literature.
